# Baseline level and change trajectory of the triglyceride-glucose index in relation to the development of NAFLD: a large population-based cohort study

**DOI:** 10.3389/fendo.2023.1137098

**Published:** 2023-05-08

**Authors:** Yaqin Wang, Jiangang Wang, Lei Liu, Pingting Yang, Shuwen Deng, Xuelian Liu, Linlin Zhao, Changfa Wang, Ying Li

**Affiliations:** ^1^ Health Management Center, The Third Xiangya Hospital, Central South University, Changsha, Hunan, China; ^2^ General Surgery Department, The Third Xiangya Hospital, Central South University, Changsha, Hunan, China

**Keywords:** triglyceride glucose index, insulin resistance, non-alcoholic fatty liver disease, risk factors, cohort study

## Abstract

**Background:**

Insulin resistance (IR) and nonalcoholic fatty liver disease (NAFLD) are closely related. The triglyceride-glucose index (TyG index) has been proposed as a new indicator of IR. It remains unclear whether the triglyceride-glucose (TyG) index is prospectively associated with incident nonalcoholic fatty liver disease (NAFLD).

**Methods:**

This large-scale study comprised 1 prospective cohort totaling 22,758 subjects without NAFLD at baseline who underwent repeated health examinations and 1 subcohort totaling 7,722 subjects with more than three visits. The TyG index was ascertained mathematically by ln (fasting triglycerides [mg/dL] × fasting glucose [mg/dL]/2). NAFLD was diagnosed by ultrasound without other concomitant liver diseases. A combinatorial Cox proportional hazard model and latent class growth mixture modeling method were used to identify the association of the TyG index and its transition trajectories with NAFLD risk.

**Results:**

During 53,481 person-years of follow-up, there were 5319 incident cases with NAFLD. Compared with those in the lowest quartile of the baseline TyG index, participants in the highest quartile had 2.52-fold (95% confidence interval, 2.21–2.86) higher odds of incident NAFLD. Similarly, restricted cubic spline analysis showed a dose–response relationship (*p* nonlinearity<0.001). Subgroup analyses showed a more significant association in the female and normal body size populations (*p* for interaction<0.001). Three distinct trajectories of changes in the TyG index were identified. Compared with the continued low group, the moderately increasing and highly increasing groups conferred 1.91-fold (1.65–2.21) and 2.19-fold (1.73–2.77) higher NAFLD risk, respectively.

**Conclusions:**

Participants with a higher baseline TyG index or a higher excessive TyG exposure were associated with an increased NAFLD risk. The findings imply that lifestyle interventions and modulation of IR might be considered to both reduce TyG index levels and prevent NAFLD development.

## Background

Nonalcoholic fatty liver disease (NAFLD) is the leading cause of chronic liver disease worldwide and was recently renamed metabolic-associated fatty liver disease (MAFLD), affecting up to approximately 25% of the global adult population (1). As has happened with adult NAFLD, the high prevalence, natural history and prognosis of fatty liver diseases in children compared with adults (2). NAFLD is widely considered the hepatic manifestation of metabolic syndrome and is correlated with metabolic derangements such as hyperglycemia, hypertriglyceridemia, obesity and hypertension (3). Recently, a consensus renamed and redefined NAFLD with “metabolic (dysfunction)-associated fatty liver disease (MAFLD)” as a more appropriate nomenclature to stress metabolic abnormalities (4). Moreover, emerging data suggest that NAFLD is not as ‘benign’ as considered and is strongly associated with an increased risk of cardiovascular diseases, extrahepatic cancers and liver-related complications (5–7). Consequently, there is a need to explore potential noninvasive markers that could be used to predict NAFLD risk to facilitate diagnosis and timely intervention at early stages to curb this emerging worldwide pandemic.

Insulin resistance (IR) and NAFLD have a very close relationship. IR is implicated not only in the pathogenesis of NAFLD but also in disease progression from steatosis to nonalcoholic steatohepatitis and fibrosis (8). Recently, the triglyceride-glucose (TyG) index, which is simple and easily calculated, has been suggested as a reliable and surrogate biomarker of IR. Growing evidence has demonstrated a pivotal role of the TyG index in mediating atherosclerotic burden and arterial stiffness progression associated with cardiovascular disease in the general population or subjects with and without diabetes (9–11). Compared with the homeostasis model assessment of IR (HOMA-IR), the TyG index shows better performance for cardiovascular predictability (12, 13).

Indeed, prior reports on the relationships of the TyG index and incident NAFLD or liver fibrosis have been explored, but there were also several limitations, so the results remain controversial (14). These previous studies were inherently limited by small sample sizes or a cross-sectional study design (15–18). Moreover, most studies used a single measurement of the baseline TyG index, which omitted the potential variability over time, and such variability may have contributed to the clinical significance of the TyG index (19–21). In some cases, the relationship was examined in females or elderly individuals only (17, 22).

In view of the aforementioned gaps in the literature, we conducted a large cohort study to capture the longitudinal TyG index dynamic changes from 2015 to 2020 and to analyze the association of NAFLD risk with both the baseline TyG index and its change trajectories derived from repeated measurements over the follow-up. In addition, we also evaluated any possible effect modifiers of the association by subgroup analysis. This study extends previous observations and provides an important clue to the development of NAFLD.

## Materials and methods

### Study design and participants

This cohort study was conducted from January 2015 to December 2020 and included participants who underwent a routine health check-up examination at the Health Management Center in the Third Xiangya Hospital of Central South University (Changsha). The study population consisted of subjects who underwent at least two health examinations that included transabdominal ultrasonography performed at least 1 year apart (n = 36,626).

**Figure 1:**
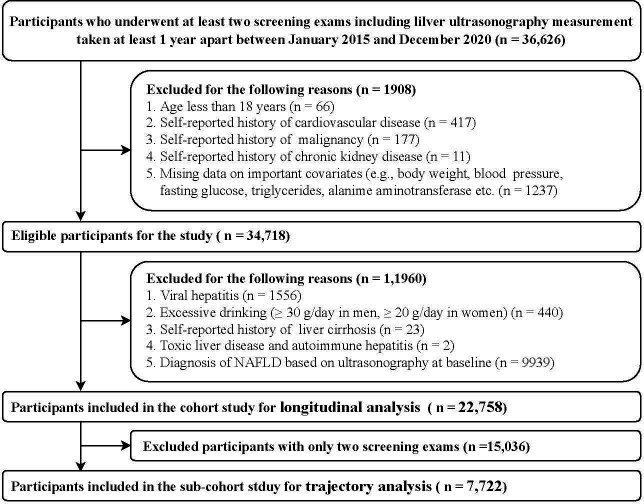
Flow diagram of study participants selection.

Participants were excluded if they had a history of cardiovascular disease, malignancy, chronic kidney disease, cirrhosis, excessive drinking, concomitant liver diseases and NAFLD at baseline (**Figure 1**). Finally, in the present study, (1) a total of 22,758 individuals in the cohort were eligible for the baseline TyG index longitudinal analysis; and (2) 7,722 individuals with more than three screening exams in the subcohort study were eligible for the TyG index changing trajectories analysis.

The study protocol was approved by the Institutional Review Board (IRB) of the Third Xiangya Hospital, Central South University (No. 2013BAI04B01). Written informed consent was obtained from all participants.

### Characteristics and definitions

Information on demographic characteristics, lifestyle factors, personal medical history was obtained from a standardized questionnaire *via* a website (https://new.selfhealth.com.cn/#/login) as previously described (23). Current smoking was coded present if more than one cigarette per day (on average) was consumed over a period longer than six months. Current drinking was coded as present if the individual reported alcohol consumption at least two days per week. Regular exercise was defined as performance of physical activities more than three times a week for thirty minutes each time. Hypertension was defined as either a self-reported history of hypertension, SBP or DBP ≥ 140/90 mmHg, or specific antihypertensive treatment (24). Diabetes was defined as self-reported history of diabetes, FBG ≥ 7.0 mmol/L, use of insulin or oral anti-diabetic drugs (25). Dyslipidemia was defined as the presence of one or more of the following: total cholesterol (TC) ≥ 6.22 mmol/L; low-density lipoprotein cholesterol (LDL-C) ≥ 4.14 mmol/L; density lipoprotein cholesterol (HDL-C) <1.04 mmol/L; triglyceride (TG) ≥ 2.26 mmol/L, or treatment for dyslipidemia (26).

Height and weight were measured by an automated instrument with participants wearing light clothing and without shoes. Body mass index (BMI) was calculated as weight in kilograms divided by height in meters squared. Waist circumference (WC) was measured from the bottom edge of the last rib and iliac crest. Blood pressure (BP) was measured using a validated digital automatic analyzer (Omron 9020). According to the Working Group on Obesity in China, BMI ≥ 24.0 kg/m² was defined as general overweight/obesity, and according to the Chinese Diabetes Society, WC ≥ 90 cm in men and ≥ 85 cm in women was defined as central obesity (27).

### Laboratory measurements

Fasting blood samples were obtained in the morning after a 12-h overnight fast. A range of biochemical parameters including fasting blood glucose (FBG), total cholesterol (TC), triglycerides (TGs), low-density lipoprotein cholesterol (LDL-C), high-density lipoprotein cholesterol (HDL-C), alanine aminotransferase (ALT), serum creatinine and uric acid, was measured using an automatic biochemical analyzer (Hitachi 7600; Hitachi, Tokyo, Japan). The sample analysis was performed in accordance with the manufacturer’s specifications. The estimated glomerular filtration rate (eGFR) was used as an index of renal disease based on the Modification of Diet in Renal Disease formula for Chinese subjects: eGFR = 175 × Scr^−1.234^ × age^−0.179^ [if female, × 0.79] (28). The TyG index was calculated as ln[fasting triglyceride (mg/dL) × fasting glucose (mg/dL)/2] (29).

### Determination of NAFLD and outcome

Fatty liver was detected by hepatic ultrasound (Logiq 9, GE Medical System, Milwaukee, WI, USA) as previously described (30). Hepatic steatosis was defined as increased hepatic echogenicity (‘bright liver’), compared to two of the three following criteria: liver-to-kidney contrast, vascular blurring and deep beam attenuation based on the Asia–Pacific Working Party recommendations (31, 32). NAFLD was defined as the presence of hepatic steatosis without excessive drinking (≥30 g/day in men, ≥20 g/day in women) or concomitant liver diseases (33).

The outcome was the incidence of NAFLD over the follow-up through December 31, 2020. If a participant had >1 event diagnosed as NAFLD during the follow-up period, the first event was counted as the outcome. Participants who did not have any NAFLD event were censored, last follow-up or December 31, 2020, whichever came first. The follow-up time was calculated using the date on which NAFLD was newly identified or to the last available follow-up visit among those who did not develop NAFLD minus the date of the baseline visit.

### Statistical analysis

Baseline characteristics are described as the mean ± standard deviation (SD) and the medians with interquartile ranges for continuous variables and as numbers (percentages) for categorical variables. Comparisons across quartiles of TyG index groups were conducted by t test or Mann−Whitney U test, and chi-square test where applicable, with specific *p* values compared with the reference group (the lowest quartile). Linear trends among groups were investigated using the Jonckheere-Terpstra test for continuous variables and the Cochran–Armitage trend test for categorical variables.

The associations between the baseline TyG index (assessed by both quartiles and as a continuous variable) and NAFLD risk were estimated in 22,758 participants as follows: (1) Cox proportional hazard regression was used to determine the hazard ratios (HRs) with corresponding 95% confidence intervals for NAFLD risk across the quartiles of the TyG index. (2) Subgroup analyses were conducted to identify interactions between TyG index quartiles and clinically relevant groups, defined by sex, age (<60 vs. ≥60 years), general overweight/obesity (yes vs. no defined by BMI) and central obesity (yes vs. no defined by WC). These grouping variables have been reported to be associated with NAFLD risk (34, 35). (3) The restricted cubic spline analysis was fitted to explore the dose−response relationship of the TyG index (continuous variables) and risk of NAFLD.

Three models were utilized for the analysis. Model 1 was a univariate analysis, model 2 was adjusted for age and sex, and model 3 was adjusted for model 2 plus multiple covariables. We followed a 2-step approach for the inclusion of covariates in the models. First, clinical covariables were entered into the univariate Cox regression analysis. Those variables that showed a *p* value < 0.10 in the univariate analyses or were selected *a priori* based on possible clinical significance were entered into the multivariable regression analyses. Second, in cases of multicollinearity, BMI was selected to replace WC, and systolic blood pressure was chosen to replace diastolic blood pressure.

Additionally, the associations between changing trajectories of the TyG index and NAFLD risk were estimated in 7,722 participants with at least three visits, which enabled us to detect the dose-accumulative association. The TyG index trajectories were identified using the latent class growth mixture modeling method, and similar change patterns were assigned to corresponding groups. Models were fitted using the LCMM package (version 1.8.1) in R (version 4.2.2, Vienna, Austria). The optimal shape of trajectories (intercept, linear, or quadratic) and the number of groups (starting with 2 groups) were determined by the following: (1) the value of the Bayesian information criterion (BIC) closest to 0 indicates the best-fitting model; (2) the smallest group had to include at least 3% of the sample; and (3) the average posterior probability (AvePP) for each group had to be more than 0.7. We described clinical characteristics by the trajectory groups. The association between TyG index changing patterns and the cumulative incidence rate of NAFLD was examined also with the Cox proportional hazard regression test.

To avoid potential bias due to participants with diabetes, the associations between the TyG index and the incidence of NAFLD were reanalyzed restricted to participants without diabetes at baseline (n = 21, 959) as a sensitivity analysis.

All statistical analyses were performed with SPSS software version 23.0 (IBM, Armonk, New York) and R software. Statistical significance was considered for a 2-tailed P <0.05.

## Results

### Clinical profile

The overall study population comprised 22,758 individuals (mean age 39.4 years; 41.5% male). The mean follow-up time was 2.35 years, and the average number of exams per person was 2.51.

Clinical and laboratory characteristics at baseline are provided in detail in **Table 1**. Compared with participants in the lowest quartile group, participants in higher TyG categories were more likely to be older, male, less educated and have more unhealthy lifestyles. Moreover, BMI, WC, systolic and diastolic blood pressure, total cholesterol, LDL-C, triglycerides, fasting blood glucose, alanine aminotransferase and uric acid increased gradually across the TyG quartiles, whereas the levels of HDL-C and estimated GFR decreased. Similarly, a higher prevalence of comorbidities, such as diabetes and hypertension, was more often present in the high TyG index groups.

**Table 1:** Study population clinical characteristics stratified by quartiles of TyG index (N = 22,758).

Characteristics	Total Population	Quartiles of TyG index				*P* for trend ^a^
		Quartile 1 (6.57 – 8.02)	Quartile 2 (8.02 – 8.36)	Quartile 3 (8.36 – 8.76)	Quartile 4 (8.76 – 12.40)	
**Prevalence, n (%)**	22758 (100.0)	5689 (25.0)	5690 (25.0)	5690 (25.0)	5689 (25.0)	
Demographic factors						
Age, years	39.4 ± 12.1	34.0 ± 9.71	38.0 ± 11.5 ^b^	40.9 ± 12.2 ^b^	44.6 ± 12.1 ^b^	< 0.001
Male sex, n (%)	9439 (41.5)	1095 (19.2)	1865 (32.8) ^b^	2716 (47.7) ^b^	3763 (66.1) ^b^	< 0.001
University degree, n (%)	16528 (72.6)	4428 (77.8)	4237 (74.5) ^b^	4034 (70.9) ^b^	3829 (67.3) ^b^	< 0.001
Lifestyle status						
Current smoker, n (%)	4203 (18.5)	328 (6.7)	761 (13.4) ^b^	1132 (19.9) ^b^	1928 (33.9) ^b^	< 0.001
Current drinker, n (%)	5341 (23.5)	761 (13.4)	1064 (18.7) ^b^	1375 (24.2) ^b^	2141 (37.6) ^b^	< 0.001
Regular exercise, n (%)	6542 (28.7)	1737 (30.5)	1672 (29.4)	1586 (27.9) ^b^	1547 (27.2) ^b^	< 0.001
Classic vascular risk factors						
BMI, kg/m^2^	22.5 ± 2.80	21.5 ± 2.27	21.9 ± 2.45 ^b^	22.8 ± 2.56 ^b^	24.3 ± 2.80 ^b^	< 0.001
WC, cm	71.5 ± 8.77	71.1 ± 6.86	74.2 ± 7.44 ^b^	77.6 ± 7.75 ^b^	82.9 ± 8.26 ^b^	< 0.001
Systolic blood pressure, mm Hg	118.1 ± 15.2	112.4 ± 12.3	115.3 ± 13.7 ^b^	119.0 ± 14.8	125.8 ± 16.4 ^b^	< 0.001
Diastolic blood pressure, mm Hg	72.4 ± 10.7	68.4 ± 8.91	70.5 ± 9.44 ^b^	72.9 ± 10.1 ^b^	77.8 ± 11.7 ^b^	< 0.001
Hypertension, n (%)	2652 (11.7)	177 (3.1)	372 (6.5) ^b^	666 (11.7) ^b^	1437 (25.3) ^b^	< 0.001
Anti-hypertensive medication, n (%)	1154 (5.1)	53 (0.9)	149 (2.6) ^b^	295 (5.2) ^b^	657 (11.5) ^b^	< 0.001
Fasting glucose, mmol/L	5.11 (4.81, 5.44)	4.90 (4.63, 5.17)	5.05 (4.79, 5.33) ^b^	5.17 (4.89, 5.47) ^b^	5.39 (5.04, 5.85) ^b^	< 0.001
Diabetes mellitus, n (%)	799 (3.5)	26 (0.5)	55 (1.0) ^b^	130 (2.3) ^b^	588 (10.3) ^b^	< 0.001
Anti-diabetes medication, n (%)	512 (2.2)	20 (0.4)	42 (0.7)	98 (1.7) ^b^	352 (6.2) ^b^	< 0.001
TC, mmol/L	4.84 ± 0.92	4.38 ± 0.74	4.69 ± 0.81 ^b^	4.96 ± 0.87 ^b^	5.33 ± 0.97 ^b^	< 0.001
Triglycerides, mmol/L	1.05 (0.76, 1.52)	0.62 (0.54, 0.70)	0.90 (0.83, 0.98) ^b^	1.25 (1.13, 1.38) ^b^	2.04 (1.72, 2.66) ^b^	< 0.001
HDL–cholesterol, mmol/L	1.45 ± 0.32	1.60 ± 0.31	1.52 ± 0.31 ^b^	1.42 ± 0.28 ^b^	1.27 ± 0.27 ^b^	< 0.001
LDL–cholesterol, mmol/L	2.77 ± 0.78	2.48 ± 0.63	2.74 ± 0.70 ^b^	2.96 ± 0.77 ^b^	2.92 ± 0.89 ^b^	< 0.001
Non- HDL cholesterol, mmol/L	3.39 ± 0.90	2.78 ± 0.64	3.17 ± 0.71 ^b^	3.54 ± 0.78 ^b^	4.06 ± 0.91 ^b^	< 0.001
Dyslipidemia, n (%)	4503 (19.8)	188 (3.3)	441 (7.8) ^b^	808 (14.2) ^b^	3066 (53.9) ^b^	< 0.001
Anti-dyslipidemia medication, n (%)	211 (0.9)	1 (0.0)	7 (0.1)	23 (0.4) ^b^	180 (3.2) ^b^	< 0.001
Emerging related factors						
ALT, U/L	17.0 (13.0, 25.0)	14.0 (11.0, 19.0)	16.0 (12.0, 22.0) ^b^	18.0 (14.0, 25.0) ^b^	23.0 (17.0, 33.0) ^b^	< 0.001
eGFR, mL/min/1.73m^2^	112.9 (98.1, 130.5)	122.0 (107.4, 139.0)	115.7 (100.7, 133.1) ^b^	109.5 (95.7, 126.3) ^b^	104.9 (91.5, 120.3) ^b^	< 0.001
Uric acid, μmol/L	351.5 ± 86.0	273.8 ± 67.7	291.3 ± 72.4 ^b^	315.3 ± 76.7 ^b^	351.5 ± 86.0 ^b^	< 0.001
**Follow-up period (years)**	2.35 (1.21)	2.38 (1.26)	2.38 (1.21)	2.35 (1.20)	2.29 (1.15) ^b^	0.013
**Times of screening exams**	2.51 (0.85)	2.58 (0.90)	2.54 (0.86) ^b^	2.52 (0.85) ^b^	2.38 (0.74) ^b^	< 0.001

Values are n (%), mean ± SD, or median (first quartile, third quartile).

a P values for trend were calculated by the Jonckheere-Terpstra test for continuous variables and by the Cochran–Armitage trend test for categorical variables.

b p<0.05 vs. Quartile 1 (reference group) by the one-way ANOVA test for continuous variables and by the chi-square test for categorical variables.

BMI, Body mass index; WC, waist circumference; TC, total cholesterol; HDL, high-density lipoprotein; LDL, low-density lipoprotein; ALT, alanine aminotransferase; eGFR, estimated glomerular filtration rate.

### Associations between the baseline TyG index and risk of NAFLD

During the 53,481.3 person-years of follow-up, NAFLD developed in 5,319 participants. The incidence rates (per 1000 person-years) of NAFLD for each of the quartiles were 26.8 for quartile 1, 58.5 for quartile 2, 105.9 for quartile 3, and 210.9 for quartile 4 participants.

First, the Cox proportional hazard model was conducted to evaluate the relationship between the baseline TyG index and the risk of NAFLD, as shown in **Table 2**. Covariables showing a *p* value < 0.10 in the univariate Cox analyses were selected in model 3 (**Supplementary Table S1**). There was a significant positive association in all three models. In fully adjusted model 3, the HRs (95% CI) for incident NAFLD comparing quartile 1, quartile 2, quartile 3 and quartile 4 participants were 1.62 (1.43–1.84), 2.10 (1.87–2.39), and 2.52 (2.21–2.86), respectively (*p* for trend, < 0.001).

**Table 2:** Risk for development of NAFLD across baseline TyG index categories (n = 22, 758).

TyG index Categories	Person-years	Incident cases	Incidence density (per 1000 person-years)	Multivariable-adjusted HR ^a^ (95% CI)		
				Model 1^b^	Model 2^c^	Model 3^d^
Quartile 1 (6.57– 8.02)	13546.5	363	26.8	1.00 (reference)	1.00 (reference)	1.00 (reference)
Quartile 2 (8.02– 8.36)	13542.0	792	58.5	2.21 (1.95– 2.50)	1.90 (1.67– 2.15)	1.62 (1.43– 1.84)
Quartile 3 (8.36– 8.76)	13376.3	1416	105.9	4.03 (3.59– 4.52)	2.97 (2.64– 3.35)	2.10 (1.87– 2.39)
Quartile 4 (8.76– 12.40)	13027.3	2748	210.9	8.23 (7.38– 9.18)	5.15 (4.59– 5.78)	2.52 (2.21– 2.86)
*p* for trend				< 0.001	< 0.001	< 0.001

TyG, Triglyceride−glucose; HR, hazards ratio; CI, confidence intervals.

a Estimated from Cox proportional hazard models and variables with p < 0.10 identified by univariate analyses were included in the multivariate model.

b Multivariable model 1 was unadjusted.

c Model 2: adjustment for age and sex.

d Model 3: model 2 plus adjustment for education level, current smoking, current drinking, body mass index, systolic blood pressure, high-density lipoprotein (HDL)-cholesterol, Non- HDL cholesterol, glomerular filtration rate, uric acid, ALT, medication of dyslipidemia, diabetes and hypertension at baseline.

Second, subgroup analyses by potential effect modifiers were performed under the same Cox proportional hazards framework as the primary analysis in **Table 3**. There were significant interactions between the TyG index and sex and body size shape on NAFLD risk (*p* for interaction < 0.001). The association was more evident in females than in males and in lean individuals than in individuals with general overweight/obesity or central obesity (*p* for trend ≤ 0.001). However, the association between the TyG index and NAFLD risk was evident in participants aged < 60 years (*p* for trend ≤ 0.001) but not in individuals aged ≥ 60 years (*p* for trend = 0.429). This relationship was not significantly different in the different age groups (*p* interaction = 0.089).

**Table 3:** Subgroup analyses for the association between baseline TyG index categories and incident NAFLD (n = 22, 758).

Subgroups	TyG index Categories					
	Quartile 1 (6.57– 8.02)	Quartile 2 (8.02– 8.36)	Quartile 3 (8.36– 8.76)	Quartile 4 (8.76– 12.40)	*p* for trend^a^	*p* for interaction
Age						0.089
Age < 60 years	1.00 (reference)	1.65 (1.45– 1.89)	2.16 (1.91– 2.45)	2.65 (2.32– 3.02)	< 0.001	
Age ≥ 60 years	1.00 (reference)	1.30 (0.74– 2.28)	1.52 (0.89– 2.58)	1.41 (0.82– 2.58)	0.429	
Sex						< 0.001
Female	1.00 (reference)	1.71 (1.42– 2.06)	2.22 (1.85– 2.68)	2.94 (2.40– 3.60)	< 0.001	
Male	1.00 (reference)	1.26 (1.06– 1.50)	1.51 (1.28– 1.77)	1.78 (1.51– 2.10)	< 0.001	
Body size defined by BMI						< 0.001
Lean/normal	1.00 (reference)	1.67 (1.41– 1.98)	2.05 (1.74– 2.43)	2.56 (2.14– 3.07)	< 0.001	
General overweight /obesity	1.00 (reference)	1.18 (0.97– 1.43)	1.49 (1.24– 1.79)	1.83 (1.52– 2.20)	< 0.001	
Body size defined by WC						< 0.001
Normal	1.00 (reference)	1.51 (1.32– 1.73)	1.84 (1.61– 2.11)	2.25 (1.95– 2.59)	< 0.001	
Central obesity	1.00 (reference)	1.31 (0.91– 1.89)	1.68 (1.21– 2.34)	1.80 (1.29– 2.50)	0.001	

a Estimated from Cox proportional hazard models.

All analyses were adjusted for age, sex, education level, current smoking, current drinking, body mass index, systolic blood pressure, high-density lipoprotein (HDL)-cholesterol, Non-HDL cholesterol, glomerular filtration rate, uric acid, ALT, medication of dyslipidemia, diabetes and hypertension at baseline when they were not the strata variables.

**Figure 2:**
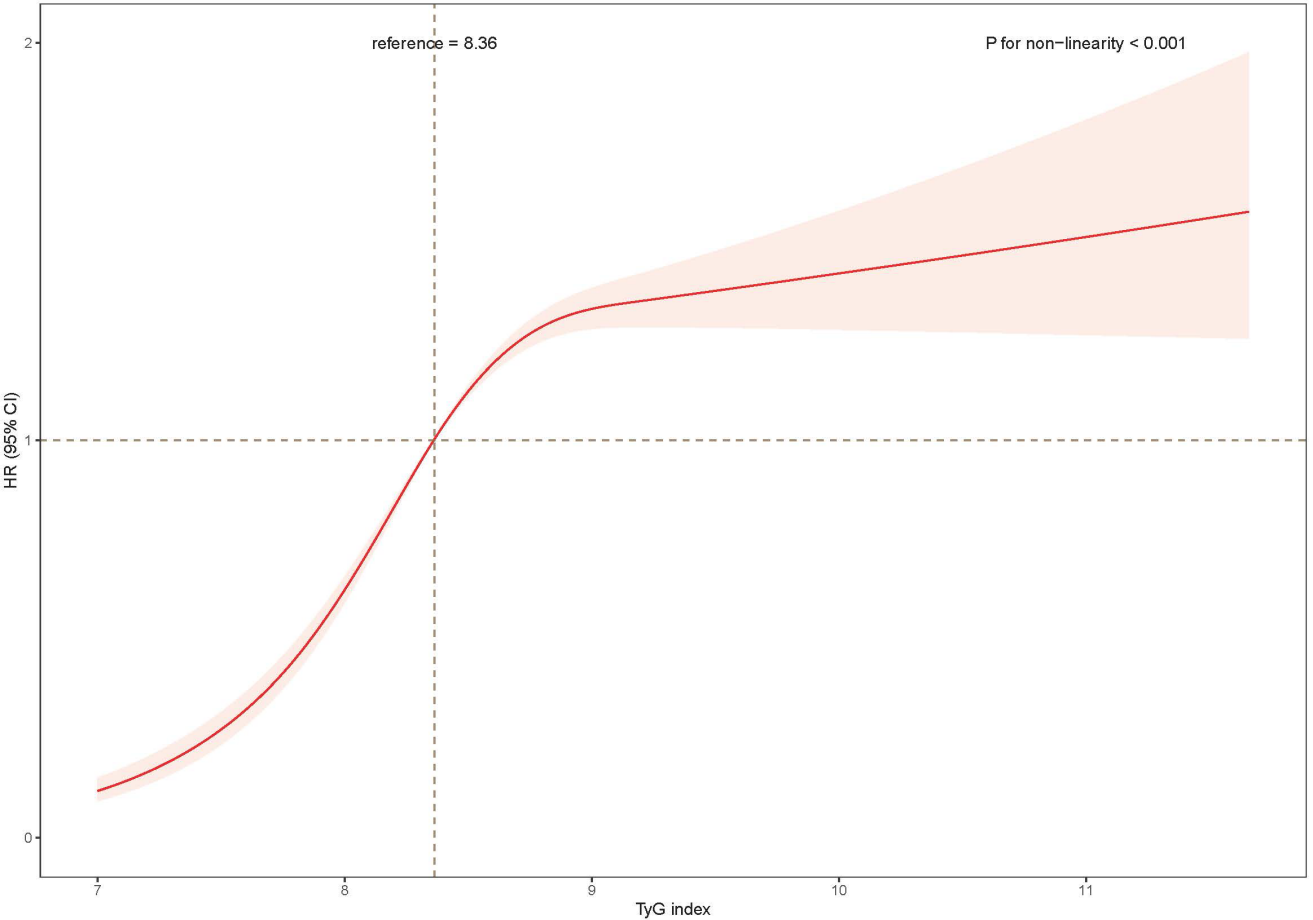
The association of the baseline TyG index with risk of NAFLD. Data were fitted using a Cox regression model of the restricted cubic spline with 3 knots at 10th, 50th, and 90th percentiles of baseline TyG index. The reference point was the median of the TyG index in the 22,758 participants. The solid line represented point estimation on the association of TyG index with the risk of NAFLD, and the shaded portion represented 95% CI estimation. Covariates in the model included age, sex, education level, current smoking, current drinking, body mass index, systolic blood pressure, high-density lipoprotein (HDL)-cholesterol, Non- HDL cholesterol, glomerular filtration rate, uric acid, ALT, medication of dyslipidemia, diabetes and hypertension at baseline. TyG, index triglyceride-glucose index; HR, hazard ratio; CI, confidence interval.

Third, the TyG index was further converted from quartiles to a continuous variable and restricted cubic spline regressions to model the associations continuously (**Figure 2**). The regression splines showed the nonlinear dose–response relationship between the TyG index and the risk of developing NAFLD (*p* nonlinearity < 0.001).

### Association between the TyG index trajectory and risk of NAFLD

**Figure 3:**
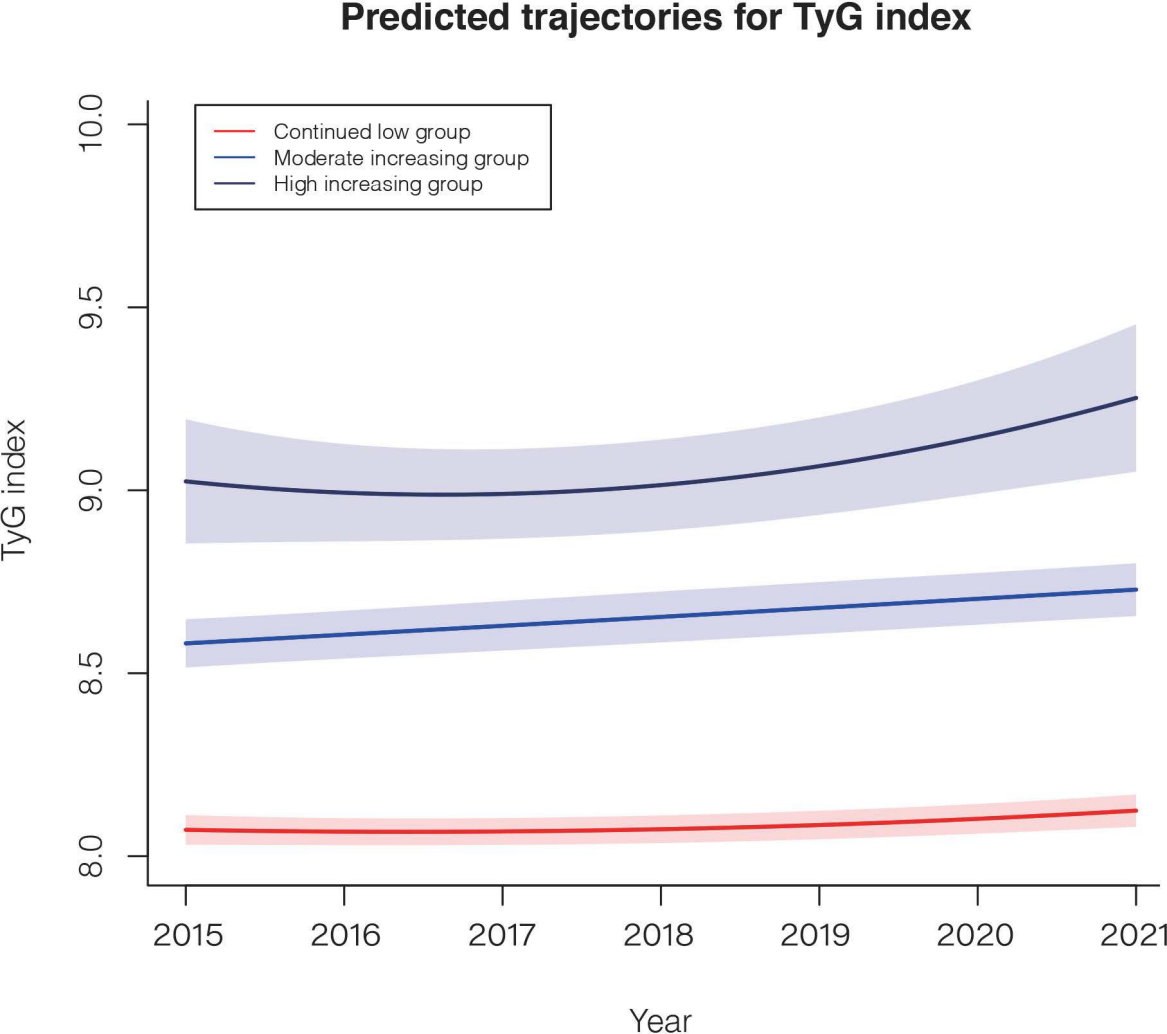
The TyG index trajectories of the 3 groups. Solid lines indicate estimated trajectories, and shaded areas indicate 95% pointwise CIs using the latent class growth mixture model.

A total of 7,722 participants were included in further trajectory analyses. The mean follow-up time was 3.20 years, and the average number of exams per person was 3.49. The optimal 3-group trajectory model was selected as the final model, and the statistical parameters for the 2-, 3-, and 4-group trajectory models are shown in **Supplementary Table S2**. Participants were classified into 3 distinct groups based on their TyG index change trajectories: continued low group (n = 4473, 57.9%), moderately increased group (n =2977, 38.6%) and highly increased group (n = 272, 3.5%) (**Figure 3**).

A comparison of the descriptive characteristics of the 3 trajectory groups is presented in **Supplementary Table S3**. Similarly, participants in the highly and moderately increasing groups were more likely to have poor lifestyles and unhealthy cardiovascular metabolic risk factors. During the 24,738.2 person-years of follow-up, there were 1349 incident NAFLD cases. Taking the continued low group as a reference, after multivariable adjustment, the HRs (95% CI) for the risk of NAFLD were 1.91 (1.65–2.21) in the moderately increasing group and 2.19 (1.73–2.77) in the highly increasing group (**Table 4**).

**Table 4:** Risk for incident NAFLD with trajectories TyG index categories (n = 7,722).

TyG index trajectories	Person-years	Incident cases	Incidence density (per 1000 person-years)	Multivariable-adjusted HR ^a^ (95% CI)		
				Model 1^b^	Model 2^c^	Model 3^d^
Continued low group	14423.6	338	23.4	1.00 (reference)	1.00 (reference)	1.00 (reference)
Moderate increasing group	9451.9	859	90.9	3.95 (3.48– 4.48)	2.80 (2.45– 3.19)	1.91 (1.65– 2.21)
High increasing group	862.3	152	176.3	7.70 (6.36– 9.32)	4.55 (3.72– 5.56)	2.19 (1.73– 2.77)
*p* for trend				< 0.001	< 0.001	< 0.001

NAFLD, non-alcoholic fatty liver disease; TyG, Triglyceride−glucose; HR, hazards ratio; CI, confidence intervals.

a Estimated from Cox proportional hazard models.

b Multivariable model 1 was unadjusted.

c Model 2: adjustment for age and sex.

d Model 3: model 2 plus adjustment for education level, current smoking, current drinking, body mass index, systolic blood pressure, high-density lipoprotein (HDL)-cholesterol, Non- HDL cholesterol, glomerular filtration rate, uric acid, ALT, medication of dyslipidemia, diabetes and hypertension at baseline.

### Sensitivity analyses

To assess the robustness of the main findings, sensitivity analysis was conducted in the participants without diabetes at baseline to reduce the possibility of relation with diabetes. Adjusted for covariates, the results showed similar positive associations between the TyG index and NAFLD risk (**Supplementary Tables S4**, **S5**).

## Discussion

In this large cohort, we observed a significant dose–response relationship between the TyG index and the development of NAFLD. In baseline TyG index variable settings, the TyG index positively correlated with NAFLD risk when considered as either a continuous variable or as quartiles. In the setting of a changing trajectory of the TyG index variable, the highly increasing trajectory carries the greatest odds of future NAFLD risk. These findings suggest a potential role for lasting high levels of IR in the pathogenesis of NAFLD. In addition, interaction effects on NAFLD risk were detected between the TyG index and sex and body size phenotype in the subgroup analysis. The associations were more evident in females than in males and in lean individuals than in overweight/obesity individuals.

A limited number of studies have demonstrated that the TyG index could play a predictive role in NAFLD. Moreover, several researchers shifted their attention to the relationship of the TyG index with the severity of hepatic steatosis and the presence of liver fibrosis (36). Two cross-sectional studies in the Chinese general population suggested that an increased TyG index was associated with a greater risk of NAFLD (17, 18). In addition, Kitae et al. and Zhang et al. observed a relationship between the baseline TyG index and incident NAFLD in a population-based cohort, indicating that a higher TyG index predicted a higher NAFLD risk among Japanese and Chinese healthy individuals (19, 21). Similarly, Zheng et al. reported that the participants in the highest TyG index quartile had a two times greater risk of incident MAFLD than those in the lowest quartile in a Chinese longitudinal study (20). Khamseh et al. also found the TyG index to be an independent risk factor for NAFLD in individuals with overweight/obesity (16). Our present results were in accordance with the few existing reports but also have a few differences. One of the most notable features is that we accounted for the dynamic changes in the TyG index and did not just take the baseline level measured at a single time point into consideration. As reported in recent studies, Gao et al. and Yan et al. found that a higher long-term trajectory of the TyG index was independently associated with incident peripheral arterial disease and arterial stiffness progression, respectively (9, 37). However, it remains unclear how the dynamic changes in the TyG index affect NAFLD risk. To our knowledge, this is the first study exploring the impact of the natural history of IR assessed by the TyG index on future NAFLD incidence. Therefore, our present results extended the limited previous observations by highlighting the dynamic nature of excessive IR exposure as it relates to increased NAFLD development. Another difference is that our study consisted of a larger sample from a population-based cohort, with diverse sociodemographic characteristics (education level and lifestyle factors). Additional sensitivity analysis including a population without diabetes adds robustness to our findings.

Although the exact mechanisms underlying the TyG index and incident NAFLD remain an enigma, the key hypothetical mechanisms may be linked to IR. IR is a key pathogenic factor for NAFLD and type 2 diabetes mellitus (T2DM), and thus, these two diseases commonly coexist. The risk of NAFLD was 5-fold higher in patients with T2DM than in those without T2DM, whereas the risk of T2DM was approximately doubled in patients with NAFLD compared with those without NAFLD (8). Several proposed mechanisms have been implicated in linking IR and the development of NAFLD (8, 38, 39): (1) IR fails to suppress both lipolysis and the production of triglyceride-rich VLDL particles through the liver. The large triglyceride-rich VLDL particles in return could exacerbate hepatic IR and increase the level of serum triglycerides. (2) IR impaired the ability of insulin to inhibit glucose production and glucose uptake, resulting in hyperglycemia, which inversely feedbacks to stimulate insulin secretion, leading to hyperinsulinemia. Fasting hyperglycemia and hyperinsulinemia are directly correlated with liver fat. (3) Additionally, IR could induce alterations in the gut microbiota to produce higher levels of short-chain fatty acids (SCFAs), modify the enterohepatic circulation of bile acids, and ultimately lead to inflammation and hepatic steatosis. (4) Moreover, the other proposed mechanisms linking IR and NAFLD may be mediated by adipose tissue inflammation, increased serum free fatty acids, impaired mitochondrial fatty acid β-oxidation (FAO), poor effects on body fat distribution, and alterations in the levels of adipokines and cytokines. The net outcomes of the above mechanisms result in an increase in chronic low-grade inflammation, a reduction in glycogen synthesis, a gain in lipogenesis, and a raising in blood insulin and glucose levels, eventually resulted in steatosis in NAFLD (8). The TyG index takes into account the effects of the two key components of metabolic syndrome, namely, fasting glucose and triglyceride, at the same time, which are overproduced by the fatty liver, and due to the advantages of being non-insulin-based, more feasible and less costly than HOMA-IR, it has become an ideal marker of IR for routine clinical applications.

Subgroup analyses revealed that the association between the TyG index and NAFLD incidence was affected by sex and body size phenotype. These results were in line with recent evidence from Zheng et al. and Kitae et al. in which the TyG index was significantly associated with a higher prevalence of incident NAFLD in a subgroup of female and lean individuals (BMI < 23 kg/m^2^), although no interactions were detected in the stratified analysis (19, 20). Our present study has further provided new insights, as key findings regarding the effect of the TyG index on NAFLD risk were more positive in female subjects than in male subjects and in lean subjects than in obese subjects, with significant interactions. The reason for the sex effect might be the age distribution of the sample. The mean age of the female participants in the current study was 38.3 years, and the sex hormone-associated protective effect in body composition and metabolic risks may explain this strong relationship in women (40, 41). The predictive capability of the TyG index in females may decrease or be similar to that in males after menopause. Similarly, the obese population tends to cluster with adverse metabolic conditions, including low HDL cholesterol levels, high blood pressure and hyperglycemia (27). It is therefore not surprising that the association between the TyG index and NAFLD risk may be partially diluted in these obese individuals by cardiometabolic risk factors. In addition, the lean population with an increased TyG index implied metabolic derangements, and this specific population was also classified as metabolically unhealthy lean people. Interestingly, compared to metabolically healthy normal weight subjects, these individuals more frequently have insulin resistance, NAFLD, visceral obesity and abnormalities in lipid storage (lower percentages of subcutaneous leg fat mass, which is the disparity of adipose tissue depot supposed to be protective of cardiometabolic diseases) (42).

The strengths of the present study were the inclusion of a large sample size, which ensures sufficient power in the investigation of interactions; adjustment to minimize residual confounders; handling target independent variables as both continuous variables and categorical variables and the potential effect modifiers by subgroup analyses. Another unique feature of our study is the use of latent class growth mixture modeling to capture distinctive TyG trajectories within the study population and to enable intraindividual variation to be investigated, thus offering a more comprehensive characterization of the natural history of the TyG index.

There are a few limitations of our study. First, the study population was restricted to the southern Chinese population and was collected at a single health management center, limiting the generalizability of the findings. Second, due to the missing records of insulin levels in our study, we could not calculate HOMA-IR and compare the TyG index with HOMA-IR for predicting incident NAFLD. Third, we did not collect detailed food intake information or eating habits. As such, we could not assess the possible modifying effect of dietary patterns on the TyG index-NAFLD association. Fourth, we performed abdominal ultrasound as a diagnostic tool for fatty liver instead of magnetic resonance imaging (MRI)/computed tomography (CT). Although ultrasound cannot accurately detect steatosis to a degree of less than 20% or in individuals with morbid and moderate obesity, due to its noninvasiveness, simplicity, and good applicability, ultrasonography has become the most common technique used in clinical practice in China to diagnose fatty liver (34). A meta-analysis showed that B-type ultrasound could reliable and accurate detect hepatic steatosis (defined as steatotic hepatocytes ≥ 5% on histology) (43). These findings showed that ultrasound was both sensitive and specific in identifying mild as well as moderate-severe hepatic steatosis, as compared with liver histology. Lastly, since fatty liver is a heterogeneous group of diseases, some researchers recommend to expand the scope of fatty liver disease (FLD) based on pathophysiology and propose the novel FLD “Mantzoros classification” (44). This new nomenclature covers alcohol-associated fatty liver disease, MAFLD, genetics-associated fatty liver disease, lipodystrophy-associated fatty liver disease and other/not yet-defined fatty liver disease. In the future, targeted the TyG index study based on this new categorization have the potential to lead to more promising merit of FLD risk evaluation.

## Conclusions

This cohort study identified a strong association between the TyG index levels and the risk of NAFLD, and the greatest predictability was seen in middle-aged adults of the female sex and normal body size populations. This association supports available evidence that IR confers higher NAFLD risk and shows that the cumulative burden of risk extends to individuals with excessive IR exposure. Thus, combinatorial approaches targeting early and sustained improvements in IR represent a potential strategy for NAFLD prevention.

## Data availability statement

The original contributions presented in the study are included in the article/**Supplementary Material**. Further inquiries can be directed to the corresponding authors.

## Ethics statement

The studies involving human participants were reviewed and approved by the Institutional Review Board (IRB) of the Third Xiangya Hospital, Central South University (No. 2013BAI04B01) and conformed to the guidelines of the Helsinki declaration and its later amendments or comparable ethical standards. The patients/participants provided their written informed consent to participate in this study. Written informed consent was obtained from the individual(s) for the publication of any potentially identifiable images or data included in this article.

## Author contributions

All authors conceived and designed the study. YW performed data analysis and drafted the manuscript. JW contributed to supervision and project administration. LL contributed to data curation. PY helped in the data analysis methods. SD contributed to software. XL and LZ collected data and ran the investigation. CW and YL contributed to the formal analysis, conception and critical revision of the manuscript. All authors read and approved the final manuscript.
