# Supplementary material for: Baseline level and change trajectory of the triglyceride-glucose index in relation to the development of NAFLD: a large population-based cohort study
Source: Front Endocrinol (Lausanne). 2023 May 8;14:1137098. doi: 10.3389/fendo.2023.1137098 (PMC10200880; doi:10.3389/fendo.2023.1137098)
Supplement: Supplementary file 1 [file Table_1.docx]

**Supplemental Material**

**Supplementary tables**

Table S1. Univariate Cox analysis between baseline TyG index quartiles and development of NAFLD risk (n = 22, 758).

Table S2. Statistical parameters for 2-, 3-, and 4- latent class growth mixture models (n = 7,722).

Table S3. Study population clinical characteristics stratified by trajectories of the TyG index (n = 7,722).

Table S4. Association of baseline TyG index with risk of NAFLD restricted in population without diabetes (n = 21, 959).

Table S5. Subgroup analyses for the association between baseline TyG index and development of NAFLD risk restricted in population without diabetes (n = 21, 959).

**Table S1. Univariate Cox analysis between baseline TyG index** **quartiles and development of NAFLD risk (n = 22, 758).**

| **Variable** | **HR (95% CI)** | **P value** |
| --- | --- | --- |
| **Demographic features** |  |  |
| Age, years | 1.03 (1.02−1.04) | <0.001 |
| females, n (%) | 0.30 (0.28−0.32) | <0.001 |
| Education, university degree, n (%) | 0.76 (0.72−0.81) | <0.001 |
| **Lifestyle factors** |  |  |
| Current smoking, n (%) | 2.08 (1.96−2.20) | <0.001 |
| Alcohol drinking, n (%) | 2.05 (1.94−2.17) | <0.001 |
| Regular exercise, n (%) | 1.03 (0.98−1.09) | 0.294 |
| **Physical measurements** |  |  |
| WC, cm | 1.09 (1.08−1.10) | <0.001 |
| BMI, kg/m^2^ | 1.27 (1.25−1.28) | <0.001 |
| Systolic blood pressure, mmHg | 1.03 (1.01−1.04) | <0.001 |
| Diastolic blood pressure, mmHg | 1.04 (1.03−1.05) | <0.001 |
| **Related risk factors** |  |  |
| Non-HDL cholesterol, mmol/l | 1.58 (1.54−1.62) | <0.001 |
| HDL cholesterol, mmol/l | 0.14 (0.12−0.16) | <0.001 |
| ALT, mmol/l | 1.03 (1.02−1.05) | <0.001 |
| eGFR, mL/min/1.73m^2^ | 0.98 (0.97− 0.99) | <0.001 |
| Uric acid, μmol/L | 1.02 (1.01− 1.04) | <0.001 |
| **Self-reported medical histories** |  |  |
| Hypertension, n (%) | 2.47 (2.22− 2.72) | <0.001 |
| Anti-hypertensive medication, n (%) | 2.45 (2.26− 2.67) | <0.001 |
| Diabetes, n (%) | 2.46 (2.22− 2.72) | <0.001 |
| Anti-diabetes medication, n (%) | 2.42 (2.13− 2.75) | <0.001 |
| Dyslipidemia, n (%) | 2.75 (2.60− 2.90) | <0.001 |
| Anti-dyslipidemia medication, n (%) | 2.52 (2.12− 3.00) | <0.001 |

Data expressed as HR (95% CI).

Abbreviations: NAFLD, non-alcoholic fatty liver disease; WC, waist circumference; BMI, body mass index; HDL, high-density lipoprotein; ALT, alanine aminotransferase; eGFR, estimated glomerular filtration rate.

**Table S2. Statistical parameters for 2-, 3-, and 4-** **latent class growth mixture models (n = 7,722).**

| **Parameter** | **Number of groups** | | |
| --- | --- | --- | --- |
|  | **2** | **3** | **4** |
| **BIC** | 26532.59 | **26343.06** | 26208.12 |
| ***N* (observed group proportion, %)** | | | |
| Group 1 | 6448 (83.5) | **4473 (57.9)** | 4333 (56.3) |
| Group 2 | 1274 (16.5) | **2977 (38.6)** | 3151 (40.8) |
| Group 3 |  | **272 (3.5)** | 184 (2.4) |
| Group 4 |  |  | 43 (0.5) |
| **AvePP** | | | |
| Group 1 | 0.84 | **0.80** | 0.78 |
| Group 2 | 0.80 | **0.75** | 0.76 |
| Group 3 |  | **0.81** | 0.80 |
| Group 4 |  |  | 0.81 |

Note: The trajectory of each group was the quartic polynomial order.

BIC: Bayesian information criterion; AvePP: Average posterior probability.

**Table S3. Study population clinical characteristics stratified by trajectories of the TyG index (n = 7,722).**

| **Characteristics** | **Total Population** | **Continued low trajectory group** | **Moderate increasing trajectory group** | **High increasing trajectory group** | ***P* value for trend ^a^** |
| --- | --- | --- | --- | --- | --- |
| **Prevalence, n (%)** | 7722 (100.0) | 4473 (57.9) | 2977 (38.6) | 272 (3.5) |  |
| **Demographic factors** |  |  |  |  |  |
| Age, years | 38.7 ± 11.6 | 35.9 ± 10.3 | 42.3 ± 12.2 ^b^ | 44.5 ± 12.6 ^b^ | < 0.001 |
| Male sex, n (%) | 2761 (38.5) | 993 (22.2) | 1572 (52.8) ^b^ | 196 (72.1) ^b^ | < 0.001 |
| University degree, n (%) | 6471 (83.8) | 3863 (86.4) | 2393 (80.4) ^b^ | 215 (79.0) ^b^ | < 0.001 |
| **Lifestyle status** |  |  |  |  |  |
| Current smoker, n (%) | 1159 (15.0) | 368 (8.2) | 685 (23.0) ^b^ | 106 (39.0) ^b^ | < 0.001 |
| Current drinker, n (%) | 1546 (20.0) | 606 (13.5) | 828 (27.8) ^b^ | 112 (41.2) ^b^ | < 0.001 |
| Regular exercise, n (%) | 2318 (30.0) | 1386 (31.0) | 870 (29.2) | 62 (22.8) ^b^ | < 0.001 |
| **Classic vascular risk factors** |  |  |  |  |  |
| BMI, kg/m^2^ | 22.1 ± 2.50 | 21.4 ± 2.29 | 22.9 ± 2.40 ^b^ | 24.2 ± 2.65 ^b^ | < 0.001 |
| Waist circumference, cm | 75.1 ± 8.00 | 72.3 ± 7.08 | 76.1 ± 7.46 ^b^ | 83.2 ± 7.91 ^b^ | < 0.001 |
| Systolic blood pressure, mm Hg | 116.2 ± 14.6 | 112.8 ± 12.6 | 120.4 ± 12.6 ^b^ | 126.0 ± 17.6 | < 0.001 |
| Diastolic blood pressure, mm Hg | 72.4 ± 10.7 | 68.4 ± 8.91 | 70.5 ± 9.44 ^b^ | 72.9 ± 10.1 ^b^ | < 0.001 |
| Hypertension, n (%) | 737 (9.5) | 193 (4.3) | 463 (15.6) ^b^ | 81 (29.8) ^b^ | < 0.001 |
| Anti-hypertensive medication, n (%) | 308 (4.0) | 72 (1.6) | 195 (6.6) ^b^ | 41 (15.1) ^b^ | < 0.001 |
| Fasting glucose, mmol/L | 5.05 (4.76, 5.37) | 4.96 (4.68, 5.25) | 5.18 (4.88, 5.50) ^b^ | 5.50 (5.13, 6.33) ^b^ | < 0.001 |
| Diabetes mellitus, n (%) | 211 (2.7) | 37 (0.8) | 116 (3.9) ^b^ | 58 (21.3) ^b^ | < 0.001 |
| Anti-diabetes medication, n (%) | 119 (1.5) | 20 (0.4) | 68 (2.3) | 31 (11.4) ^b^ | < 0.001 |
| TC, mmol/L | 4.78 ± 0.89 | 4.54 ± 0.77 | 5.10 ± 0.92 ^b^ | 5.33 ± 0.97 ^b^ | < 0.001 |
| Triglycerides, mmol/L | 1.01 (0.75, 1.44) | 0.80 (0.64, 0.96) | 1.48 (1.20, 1.88) ^b^ | 3.14 (2.14, 4.46) ^b^ | < 0.001 |
| HDL–cholesterol, mmol/L | 1.49 ± 0.31 | 1.57 ± 0.31 | 1.38 ± 0.28 ^b^ | 1.20 ± 0.31 ^b^ | < 0.001 |
| LDL–cholesterol, mmol/L | 2.74 ± 0.76 | 2.58 ± 0.66 | 2.99 ± 0.81 ^b^ | 2.51 ± 0.91 ^b^ | < 0.001 |
| Non- HDL cholesterol, mmol/L | 3.30 ± 0.87 | 2.97 ± 0.70 | 3.72 ± 0.86 ^b^ | 4.14 ± 0.96 ^b^ | < 0.001 |
| Dyslipidemia, n (%) | 1220 (15.8) | 227 (5.1) | 777 (26.1) ^b^ | 216 (79.4) ^b^ | < 0.001 |
| Anti-dyslipidemia medication, n (%) | 61 (0.8) | 2 (0.0) | 37 (1.2) | 22 (8.1) ^b^ | < 0.001 |
| **Emerging related factors** |  |  |  |  |  |
| ALT, U/L | 17.0 (12.0, 23.0) | 15.0 (11.5, 20.0) | 20.0 (15.0, 27.0) ^b^ | 24.0 (18.0, 33.0) ^b^ | < 0.001 |
| eGFR, mL/min/1.73m^2^ | 114.6 (99.3, 132.4) | 119.9 (104.8, 137.9) | 107.1 (94.0, 123.4) ^b^ | 105.6 (97.8, 122.9) ^b^ | < 0.001 |
| Uric acid, μmol/L | 299.2 ± 79.1 | 277.7 ± 69.6 | 325.9 ± 80.1 ^b^ | 361.3 ± 92.8 ^b^ | < 0.001 |
| **Follow-up period (years)** | 3.20 (1.13) | 3.22 (1.14) | 3.18 (1.13) | 3.17 (1.10) | 0.063 |
| **Times of screening exams** | 3.49 (0.80) | 3.50 (0.80) | 3.49 (0.80) | 3.41 (0.71) | 0.222 |

Values are n (%), mean ± SD, or median (first quartile, third quartile).

^a^ *P* values for trend were calculated by the Jonckheere-Terpstra test for continuous variables and by the Cochran–Armitage trend test for categorical variables.

^b^ *p*<0.05 vs. Quartile 1 (reference group).

Abbreviations: TC, total cholesterol; HDL, high-density lipoprotein; LDL, low-density lipoprotein; ALT, alanine aminotransferase; eGFR, estimated glomerular filtration rate.

**Table S4. Association of baseline TyG index with risk of NAFLD restricted in population without diabetes (n = 21, 959).**

| **TyG index Categories** | **Person-years** | **Incident**  **cases** | **Incidence density**  **(per 1000 person-years)** | **Multivariable-adjusted HR ^a^ (95% CI)** | | |
| --- | --- | --- | --- | --- | --- | --- |
|  |  |  |  | **Model 1^b^** | **Model 2^c^** | **Model 3^d^** |
| Quartile 1 (6.57– 8.01) | 13070.8 | 344 | 26.3 | 1.00 (reference) | 1.00 (reference) | 1.00 (reference) |
| Quartile 2 (8.01– 8.34) | 13035.4 | 734 | 56.3 | 2.17 (1.91– 2.46) | 1.85 (1.63– 2.11) | 1.59 (1.40– 1.81) |
| Quartile 3 (8.34– 8.73) | 12935.3 | 1294 | 100.0 | 3.88 (3.44– 4.37) | 2.85 (2.52– 3.22) | 2.01 (1.77– 2.28) |
| Quartile 4 (8.73– 11.79) | 12630.0 | 2530 | 200.3 | 7.91 (7.07– 8.86) | 4.95 (4.40– 5.57) | 2.46 (2.15– 2.80) |
| *p* for trend |  |  |  | < 0.001 | < 0.001 | < 0.001 |

TyG, Triglyceride‑glucose; HR, hazards ratio; CI, confidence intervals.

^a^ Estimated from Cox proportional hazard models and variables with p < 0.10 identified by univariate analyses were included in the multivariate model.

^b^ Multivariable model 1 was unadjusted.

^c^ Model 2: adjustment for age and sex.

^d^ Model 3: model 2 plus adjustment for education level, current smoking, current drinking, body mass index, systolic blood pressure, high-density lipoprotein (HDL)-cholesterol, Non- HDL cholesterol, glomerular filtration rate, uric acid, ALT, medication of dyslipidemia and hypertension at baseline.

**Table S5.** **Subgroup analyses for the association between baseline TyG index and development of NAFLD risk restricted in population without diabetes (n = 21, 959).**

| **Subgroups** | **TyG index Categories** | | | |  |  |
| --- | --- | --- | --- | --- | --- | --- |
|  | **Quartile 1**  **(6.57– 8.01)** | **Quartile 2**  **(8.01– 8.34)** | **Quartile 3**  **(8.34– 8.73)** | **Quartile 4**  **(8.73– 11.79)** | ***p* for trend^a^** | ***p* for interaction** |
| Age |  |  |  |  |  | 0.194 |
| Age < 60 years | 1.00 (reference) | 1.63 (1.42– 1.86) | 2.06 (1.81– 2.35) | 2.59 (2.26– 2.96) | < 0.001 |  |
| Age ≥ 60 years | 1.00 (reference) | 1.38 (0.76– 2.53) | 1.64 (0.93– 2.91) | 1.50 (0.83– 2.69) | 0.176 |  |
| Sex |  |  |  |  |  | < 0.001 |
| Female | 1.00 (reference) | 1.70 (1.40– 2.06) | 2.13 (1.76– 2.57) | 3.01 (2.45– 3.70) | < 0.001 |  |
| Male | 1.00 (reference) | 1.25 (1.04– 1.49) | 1.46 (1.24– 1.73) | 1.72 (1.45– 2.04) | < 0.001 |  |
| Body size defined by BMI |  |  |  |  |  | < 0.001 |
| Lean/normal | 1.00 (reference) | 1.68 (1.41– 2.00) | 2.03 (1.71– 2.41) | 2.63 (2.19– 3.16) | < 0.001 |  |
| General overweight /obesity | 1.00 (reference) | 1.15 (0.94– 1.41) | 1.39 (1.16– 1.68) | 1.72 (1.42– 2.08) | < 0.001 |  |
| Body size defined by WC |  |  |  |  |  | < 0.001 |
| Normal | 1.00 (reference) | 1.51 (1.31– 1.74) | 1.80 (1.57– 2.07) | 2.24 (1.94– 2.59) | < 0.001 |  |
| Central obesity | 1.00 (reference) | 1.29 (0.88– 1.89) | 1.58 (1.12– 2.22) | 1.73 (1.23– 2.44) | 0.004 |  |

^a^ Estimated from Cox proportional hazard models.

All analyses were adjusted for age, sex, education level, current smoking, current drinking, body mass index, systolic blood pressure, high-density lipoprotein (HDL)-cholesterol, Non-HDL cholesterol, glomerular filtration rate, uric acid, ALT, medication of dyslipidemia and hypertension at baseline when they were not the strata variables.
